# Exploring Women’s Perceptions of Traditional Mammography and the Concept of AI-Driven Thermography to Improve the Breast Cancer Screening Journey: Mixed Methods Study

**DOI:** 10.2196/64954

**Published:** 2025-09-10

**Authors:** Kristýna Sirka Kacafírková, Anneleen Poll, An Jacobs, Antonella Cardone, Juan-Jose Ventura

**Affiliations:** 1 imec-SMIT, Vrije Universiteit Brussel, Pleinlaan 9, Brussels, 1050, Belgium, 32 026148540; 2 Cancer Patients Europe, Rue de l’Industrie 24, Brussels, 1000, Belgium

**Keywords:** breast neoplasms, early detection of cancer, mammography, thermography, artificial intelligence, patient participation, health communication, health knowledge, attitudes, practice, patient-centered care, women's health

## Abstract

**Background:**

Breast cancer is the most common cancer among women and a leading cause of mortality in Europe. Early detection through screening reduces mortality, yet participation in mammography-based programs remains suboptimal due to discomfort, radiation exposure, and accessibility issues. Thermography, particularly when driven by artificial intelligence (AI), is being explored as a noninvasive, radiation-free alternative. However, its acceptance, reliability, and impact on the screening experience remain underexplored.

**Objective:**

This study aimed to explore women’s perceptions of AI-enhanced thermography (ThermoBreast) as an alternative to mammography. It aims to identify barriers and motivators related to breast cancer screening and assess how ThermoBreast might improve the screening experience.

**Methods:**

A mixed methods approach was adopted, combining an online survey with follow-up focus groups. The survey captured women’s knowledge, attitudes, and experiences related to breast cancer screening and was used to recruit participants for qualitative exploration. After the focus groups, the survey was relaunched to include additional respondents. Quantitative data were analyzed using SPSS (IBM Corp), and qualitative data were analyzed in MAXQDA (VERBI software). Findings from both strands were synthesized to redesign the breast cancer screening journey.

**Results:**

A total of 228 valid survey responses were analyzed. Of 228, 154 women (68%) had previously undergone mammography, while 74 (32%) had not. The most reported motivators were belief in prevention (69/154, 45%), invitations from screening programs (68/154, 44%), and doctor recommendations (45/154, 29%). Among nonscreeners, key barriers included no recommendation from a doctor (39/74, 53%), absence of symptoms (27/74, 36%), and perceived age ineligibility (17/74, 23%). Pain, long appointment waits, and fear of radiation were also mentioned. In total, 18 women (mean age 45.3 years, SD 13.6) participated in 6 focus groups. Participants emphasized the importance of respectful and empathetic interactions with medical staff, clear communication, and emotional comfort—factors they perceived as more influential than the screening technology itself. ThermoBreast was positively received for being contactless, radiation-free, and potentially more comfortable. Participants described it as “less traumatic,” “easier,” and “a game changer.” However, concerns were raised regarding its novelty, lack of clinical validation, and data privacy. Some participants expressed the need for human oversight in AI-supported procedures and requested more information on how AI is used. Based on these insights, an updated screening journey was developed, highlighting improvements in preparation, appointment booking, privacy, and communication of results.

**Conclusions:**

While AI-driven thermography shows promise as a noninvasive, user-friendly alternative to mammography, its adoption depends on trust, clinical validation, and effective communication from health care professionals. It may expand screening access for populations underserved by mammography, such as younger and immobile women, but does not eliminate all participation barriers. Long-term studies and direct comparisons between mammography and thermography are needed to assess diagnostic accuracy, patient experience, and their impact on screening participation and outcomes.

## Introduction

### Background

Breast cancer remains the most common type of cancer and the leading cause of death among European women [1]. In 2022, it was responsible for nearly one-third of all new cancer cases among women in 27 European countries [2]. Early cancer detection through screening is a key part of a cancer prevention strategy [3]. Screening programs are therefore developed to establish guidelines prioritizing who should be invited based on age and risk factors [4]. These guidelines consider cost-effectiveness and balance the trade-off between the harms of overdiagnosis and the reduction of mortality due to early detection [5].

Despite free access to cancer screening programs in many European countries, this offer is not taken up by all women among the target population [6]. The decision to attend screening is influenced by a mix of habitual, practical, and emotional factors, including past health care experiences, logistical barriers, and fear of pain or radiation [7]. Awareness, trust in health care professionals, and social support are important motivators, while lack of information, low perceived risk, and discomfort serve as common barriers [8-10].

Concerns about discomfort and radiation exposure associated with mammography are one of the major barriers [11], leading to an ongoing search for alternative screening technologies. This study explores the potential of thermography as a new screening method by examining the barriers in current breast cancer screening, gathering women’s perceptions of thermography, and identifying ways to improve the screening journey to encourage higher participation.

### Current Screening Techniques and Their Limitations

Several breast cancer methods are currently available. The most commonly used is mammography, with digital breast tomosynthesis (DBT) emerging as a new diagnostic tool [12]. Second, breast ultrasound has also progressed beyond its initial role in differentiating cysts versus solid masses [13]. Third, breast magnetic resonance imaging (MRI) is the standard supplemental screening tool available for both high and intermediate-risk women [14].

Each of these techniques presents advantages and comes with some limitations. Ultrasound is commonly used as a supplemental screening technique for women with dense breasts in whom mammography has a lower sensitivity. However, ultrasound has been criticized for its relatively low specificity, leading to many recalls and biopsies for benign lesions [15]. Breast MRI offers the highest sensitivity for detecting occult cancer, regardless of breast density [14]. Nevertheless, high costs and the limited tolerability and availability of MRI scanners make population-wide screening difficult [16]. Mammography, despite its proven accuracy in detecting early-stage cancers, presents challenges such as radiation exposure, discomfort, and reduced effectiveness in women with dense breast tissue [17-19].

### Thermography as an Emerging Alternative

Given the limitations of conventional screening methods, researchers have explored alternative technologies, such as thermography. Thermography is a noninvasive imaging technique that detects heat patterns and blood flow abnormalities, which may indicate malignancy [20]. Unlike mammography, it does not involve radiation exposure or physical compression of the breast, making it a potentially more comfortable and accessible option for screening [17,18,21,22]. Nonetheless, its effectiveness in early-stage cancer detection remains debated due to lower specificity and higher false-positive rates, making it as yet unsuitable to fully replace mammography for early cancer detection [18,19,23].

Advancements in artificial intelligence (AI) have significantly improved thermography’s diagnostic potential [24]. AI algorithms can enhance image analysis, detect subtle thermal abnormalities, and improve sensitivity [21-23,25]. Recent studies have shown promising results, suggesting that AI-driven thermography could complement mammography in screening programs [22,23,25]. However, further research is needed to validate its clinical applicability and reliability [18,21,23]. Given the persistent barriers associated with current screening methods and the emerging promise of AI-enhanced thermography, it is essential to understand how potential users perceive this technology. Exploring these perceptions can offer insights into how new screening options may better meet women’s needs and expectations.

### Goal of the Study and Contribution

This study aims to explore potential gaps in the current screening journey, from the decision to undergo screening through the procedure itself to receiving results, by listening to women’s perspectives. In doing so, we aim to identify key barriers, improve the user experience, and assess whether AI-driven thermography can serve as a more accessible and less intimidating alternative.

Several studies have explored the experiences of women in breast cancer screening. For example, Ciria-Suarez et al [26] have mapped the breast cancer patient journey, highlighting areas where patient experience can be improved. Other studies, such as those by MacKinnon et al [27] and Foerster et al [28] have examined disparities in access to screening and the psychological factors influencing participation. In addition, Kim et al [29] have explored how cultural and linguistic factors influence screening participation among Korean women in Australia, demonstrating that specific barriers exist for different population groups. This focus on minorities can also be found in Racine and Andsoy’s study [30]. While these studies provide valuable insights, they primarily focus on the broader health care journey and mammography experiences.

Our study differs by specifically examining the user experience of AI-driven thermography. While previous research on thermography has concentrated on technical aspects and diagnostic accuracy [17], our work focuses on screeners’ perceptions, trust, and acceptance of this novel screening method. By incorporating feedback on the ThermoBreast procedure, we aim to understand how this approach could address barriers associated with traditional mammography and improve breast cancer screening accessibility.

## Methods

### Study Context: ThermoBreast Project

This study was conducted as part of the EU-funded ThermoBreast project, which aims to develop an advanced thermography device as a more comfortable, accessible, and potentially equally precise alternative to mammography [31]. At the start of the project, an animated video [32] was created to illustrate the ThermoBreast procedure. This visual aid was used to explore women’s screening preferences and to inform strategies for increasing participation in breast cancer screening programs.

**Figure 1.**
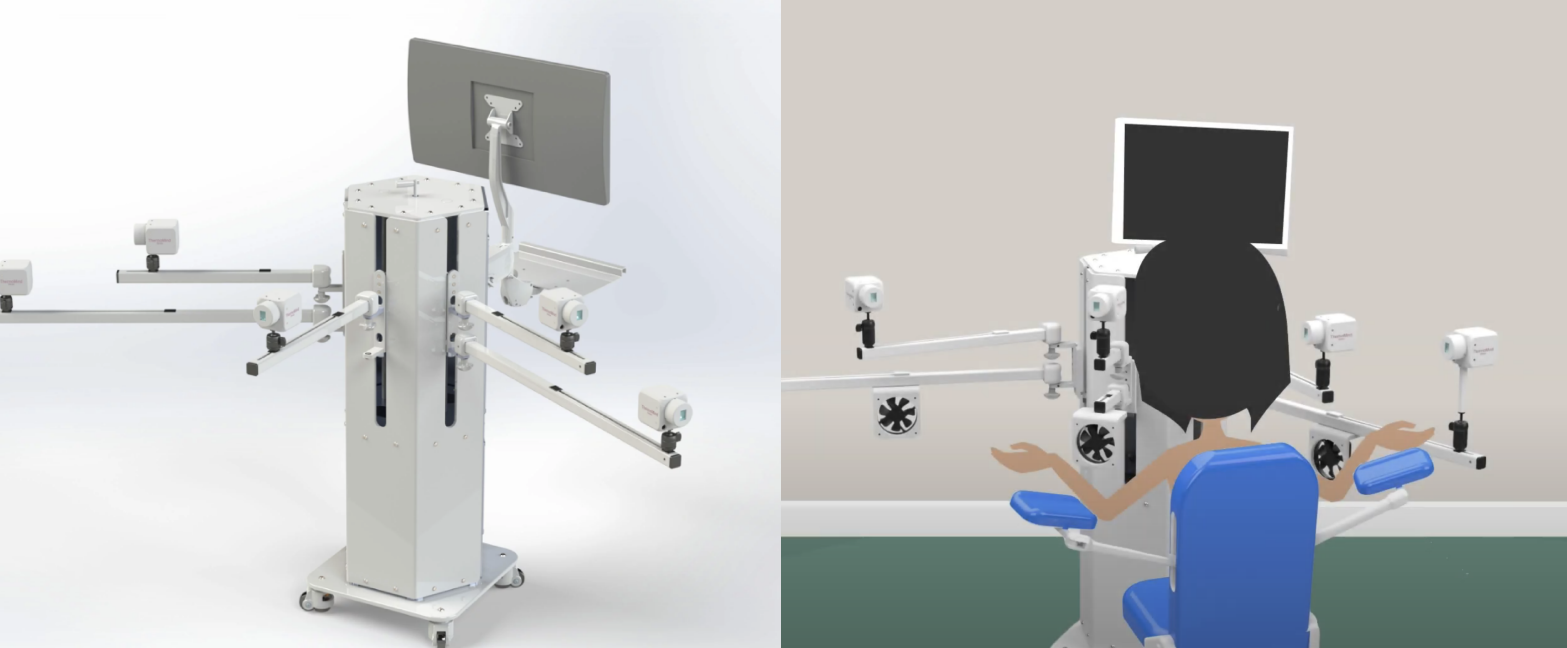
ThermoBreast screening device as shown in the animated video.

The AI-based thermography system (Figure 1) featured in the video was still in preparation for clinical trials at the time of the study and had not yet been approved. It combines medical thermography, using highly sensitive infrared cameras capable of detecting temperature differences as small as 0.02°C, with an AI model that analyzes dynamic thermal patterns to detect vascular abnormalities associated with malignancy [31].

During the procedure, the participant sits in a chair with armrests and opens their arms while cameras scan the chest and underarm regions. The process is entirely contact-free and takes approximately 12 minutes. Due to its noninvasive and inclusive design, which does not impose age or breast size restrictions, the technology has the potential to address key limitations of mammography, especially for individuals for whom the procedure is not recommended or who view it negatively.

### Study Design and Overview

**Figure 2.**
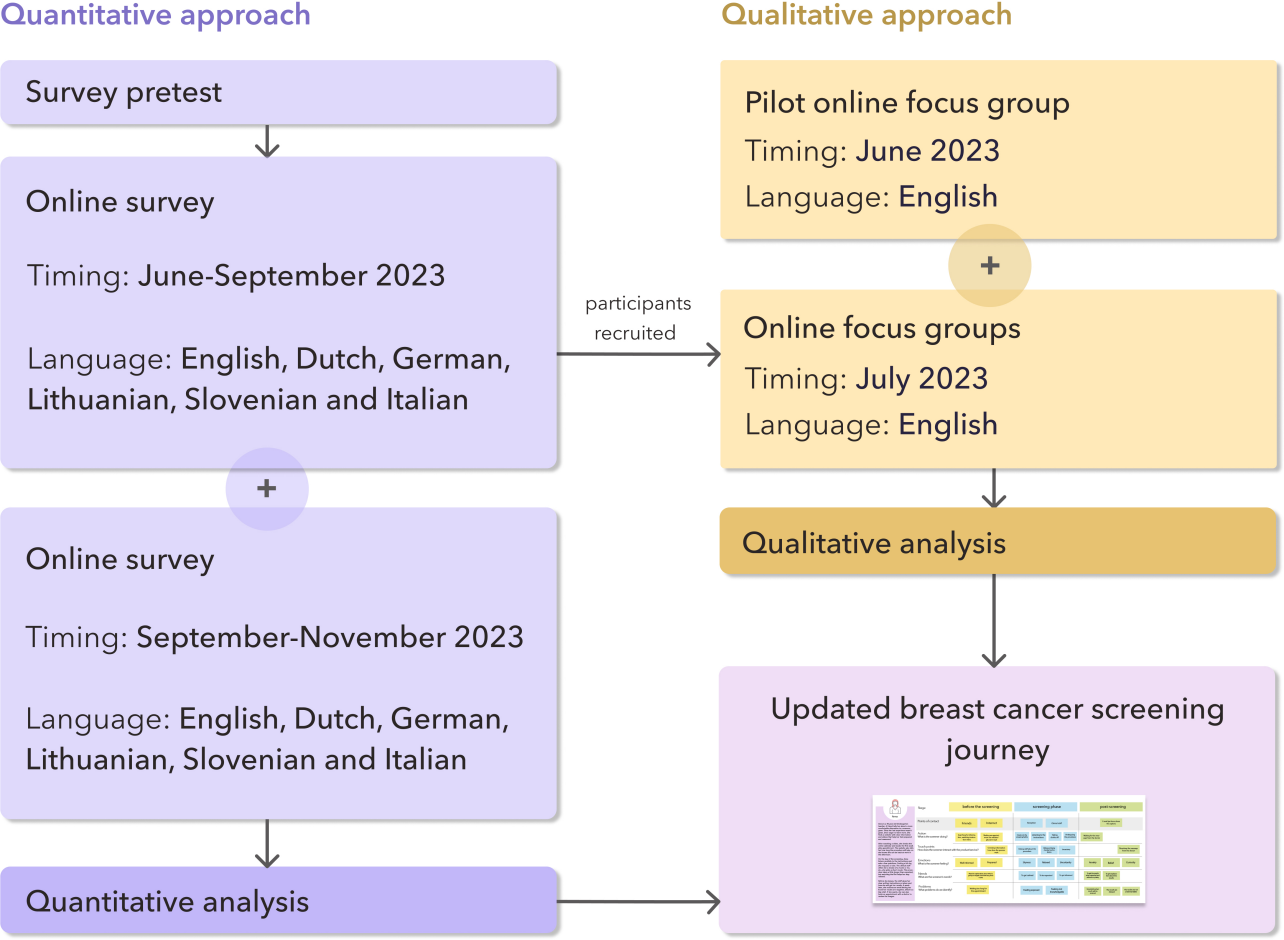
Study design overview.

To gain both broad insights into and in-depth feedback on the ThermoBreast procedure, we adopted a mixed method approach combining an online survey with follow-up focus groups. The survey served a dual purpose: collecting initial quantitative data and recruiting participants for the focus groups. After the focus groups were completed, the survey was relaunched to gather additional responses from new participants. The study timeline is presented in Figure 2. Insights from both methods were used to update and visually map the breast cancer screening journey.

### Eligibility Criteria

To participate in the survey, respondents had to be female, at least 18 years old, and understand one of the following languages: English, Dutch, German, Lithuanian, Slovenian, or Italian. For the focus groups, participants were required to understand English to enable cross-European participation and ensure a diversity of perspectives.

### Questionnaire Development

The questionnaire was developed by authors [KSK and AJ] based on previous studies [8,9,33] and consisted of 24 items. It addressed attitudes, awareness, and knowledge [8,9]; the role of social networks and health care providers [9]; personal and sociocultural factors; and cues to action [33] that influence motivators and barriers to mammography. Sociodemographic questions were also included. To ensure relevance, the questions were discussed in detail with a project partner from a cancer organization (co-author [AC]). The final version was reviewed by other project partners, including medical professionals, who also contributed to the translation process.

The questionnaire included multiple-choice items, open-ended questions, and items rated on a 5-point Likert scale (refer to Multimedia Appendix 1 for the full list of questions). It was pretested internally for timing, clarity, and technical functionality by several project partners and imec-SMIT researchers who were not involved in its development. Based on their feedback, minor adjustments were made. The final version took approximately 10 minutes to complete.

The original questionnaire was developed in English and translated into Dutch, German, Lithuanian, Slovenian, and Italian by project partners. The selected languages reflect the countries represented in the project consortium and the partners’ ability to effectively distribute the survey within their local contexts.

### Online Survey Recruitment

The survey was distributed through a social media post created by our project partner, Accelopment, which included a direct link to the Qualtrics survey (refer to Multimedia Appendix 2 for the message and post). It was shared across the ThermoBreast project’s social media channels, including Facebook, WordPress, and LinkedIn, and was also reposted by project members and partners, such as accelopment and Cancer Patients Europe (CPE). In addition, the link was circulated through an email campaign by CPE partners [AC and JJV] via their newsletter and network, and it was published on both the ThermoBreast and CPE websites.

Given these distribution channels—especially LinkedIn, the ThermoBreast website, and the CPE website—the sample likely skews towards individuals with higher education levels or interests in health-related topics. The CPE website, in particular, reaches out to cancer survivors and patients. Therefore, the sample should be regarded as a convenience sample, influenced by the nature of these dissemination contexts.

### Online Survey Data Collection

Data collection was carried out using the online platform Qualtrics (Silver Lake), which automatically recorded responses. Questions were adapted dynamically based on participants’ indicated experience with breast cancer screening: some were shown only to women with previous experience, others only to those without. Participants were provided with the option to review and revise their responses using the Back button. The survey was open from 26 June to November 7, 2023. Preliminary results were analyzed in early September. After the focus groups held in July, the question about optional participation was removed. The survey was accessible without a password, and no direct contact with potential participants occurred beforehand. Participation required agreement to informed consent and compliance with eligibility criteria. Response completeness was monitored automatically by Qualtrics, and IP addresses were collected to identify the number of unique respondents.

### Topic Guide Development

The semistructured topic guide (Multimedia Appendix 1) was developed by authors [KSK and AJ] in consultation with the CPE organization and informed by themes from previous literature [29,34] and the animated ThermoBreast video, to ensure all relevant screening-related topics were addressed. Topics included past screening experiences, first impressions of ThermoBreast, perceived comfort, the role of AI and trust in technology, and communication of results. The draft was reviewed with coauthor [AC] and pilot tested in a focus group with 7 female experts in human-computer interaction and digital health from Vrije Universiteit Brussel. This session, moderated by authors [KSK with AP] as note-taker, was used to evaluate group norms (eg, “raise hand” function), platform functionality, and question flow. Based on feedback from this session and feasibility considerations, minor refinements were integrated into the final guide.

### Online Focus Groups Recruitment

At the end of the survey, participants could express interest in joining focus groups by providing their contact details and indicating a participation slot. These individuals were later invited via email by the researcher [KSK] by sending them a link to Microsoft Teams and an informed consent form. Experts participating in the pilot focus groups were recruited directly by researchers through their professional network.

### Online Focus Group Data Collection

Focus groups were conducted online via Microsoft Teams, each lasting 60 minutes. Sessions were recorded with participants’ previous written and verbal consent. A moderator [KSK or AP] led the discussion, while a note-taker [AP or KSK] documented key points.

### Statistical Analysis

Descriptive statistics, including frequencies and cross-tabulations, were conducted. Reliability of scale-based questions was assessed using Cronbach α. *t* tests were performed for selected comparisons. All analyses were conducted by KSK using SPSS (version 29.0, IBM Corp). Open-ended responses were extracted, coded in Microsoft Excel, and integrated into the SPSS dataset by KSK. Responses from ineligible participants (eg, incomplete responses, “male” and “other” participants, try-out responses, etc) were excluded from the analysis.

### Qualitative Data Analysis

Microsoft Teams auto-generated transcripts were reviewed and edited as needed by researchers [KSK and AP]. Although initially intended as a pilot, the expert focus group provided relevant thematic insights and was included in the final coding and analysis. All names were pseudonymized. Transcripts were coded in MAXQDA 2022 using open, axial, and selective coding [35] by 2 white female researchers [KSK and AP] of European origin. To ensure reflexivity, initial coding was performed independently, followed by discussions to align interpretations and finalize the coding scheme. Final themes were defined, named, and visualized to enhance transparency (Results section).

### Revision of Breast Cancer Patient Journey

Themes, selected focus group quotes, and key survey insights were organized on a Miro board and restructured to reflect the stages of the breast cancer screening journey. This material informed the update of a reference journey map from [36], following journey mapping principles [37]. The outline was created in Miro board and finalized in Adobe Illustrator [KSK]. Several personas were drafted based on both qualitative and quantitative data by KSK and AP, and the one most closely aligned with the European screening target group (age 45‐49 years) [38] was selected for the final visualization and detailed patient journey.

### Ethical Considerations

Ethical approval (number 14320023000111) was obtained on June 22, 2023, from the Health Research Ethics Board of University Hospital Brussels (UZ Brussels). All participants received written informed consent forms detailing study purpose, procedures, duration, risks and benefits, right to withdraw, and privacy safeguards. Survey participation required agreement via an “I understand and agree” button. Data were anonymized via the encrypted Qualtrics platform. Email addresses collected for focus group invitations were stored separately and deleted from Microsoft SharePoint after study completion.

Informed consent forms for focus groups were sent to participants by email and returned signed. Sessions were recorded with previous permission and transcribed using Microsoft Teams. Transcripts were checked and corrected against the recordings [KSK and AP], and names were pseudonymized (P=participant and E=expert). No monetary or nonmonetary compensation was provided for participation in either the survey or focus groups.

## Results

### Focus Group Results

#### Data Collection and Participants

The online focus groups explored participants’ past breast screening experiences, motivations, and perceptions of thermography. The pilot consisted of 1 group with 7 women aged 25‐48 years (mean 36.7, SD 7.8 years). Two women had experience with screening, and 5 participants were nonscreeners. Out of 101 survey respondents, 24 expressed interest in joining the focus groups and provided their contact information. All interested individuals were contacted; however, only 11 chose to participate. The online focus groups with potential screeners contained data of 11 women aged 25‐66 years (mean 50.7, SD 13.8 years) who participated in 5 different groups. For more details of the participants, please consult Table 1 below.

**Table 1.** List of participants in the online focus groups.

Focus group and participant	Nationality	Age (y)	Breast cancer screening or test experience
Pilot			
E1^a^	Dutch	32	None, but breast cancer in the family
E2	Brazilian	33	Clinical examination; mammography experience heard from her mother
E3	Belgian	48	MRI^b^, mammography, ultrasound
E4	Dutch	25	None; experience heard from the family
E5	Dutch	37	None
E6	Belgian	45	None
E7	Belgian	37	None, but breast cancer in the family
1			
P1^c^	Belgian	57	Regular mammography screener, breast cancer in the family
P2	Dutch	49	BC^d^ patient; various screening experiences: mammography, ultrasound, tomosynthesis, MRI
2			
P3	US	63	MRI, mammography
3			
P4	Montenegrin	25	*BRCA* gene; ultrasound, MRI
P5	UK	49	BC patient; mammography, MRI, ultrasound
4			
P6	N/A	54	*BRCA* gene; mammography, MRI, ultrasound
P7	Spanish	25	Clinical examination
P8	Dutch	55	*BRCA* gene; mammography
5			
P9	N/A	62	Mammography
P10	Spanish	66	History of BC; regular screener
P11	Irish	53	Mammography

a E: expert.

b MRI: magnetic resonance imaging.

c P: participant.

d BC: breast cancer

### Themes

**Figure 3.**
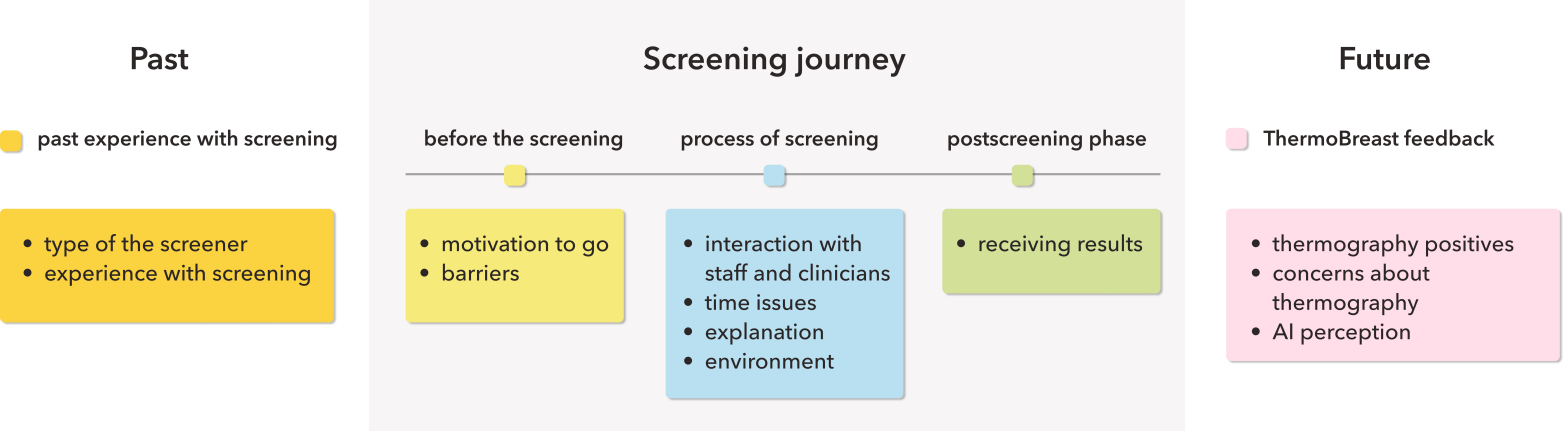
Coding scheme based on the focus groups data.

The coding process resulted in 5 main categories: past experience with screening, before the screening, the screening process, the postscreening phase, and thermography feedback. The past experience category explores women’s previous experiences with any type of screening. The screening journey category focuses on factors that could be improved in screening with thermography, based on the video shown and experiences from different breast cancer screening methods. The final category examines opinions on the concept of thermography technology and the use of AI. Refer to Figure 3 for an overview of the coding scheme.

### Past Experience With Screening

Most of the participants did have experience with some kind of screening or a breast-cancer test procedure (such as mammography, MRI, ultrasound, and tomography). Some participants (P2, P5, P6, P9, and P11) shared problems with discomfort and pain during the mammography, described as: “squeezing,” “smashing,” and “panini experience.” A few participants also mentioned their concerns about using x-rays and their willingness to understand why this is needed. More discomfort and “traumatic experience” were assigned to MRI, which was mentioned by 4 participants (E3, P2, P4, and P5). On the other hand, ultrasound was described several times as the most comfortable method. Overall, almost all participants were positive about the idea of screening and found it important.

#### Before the Screening Phase

Participants’ motivations for screening included: a family history of breast cancer or genetic predisposition (P1, P4, P8, E1, and E7), feeling of responsibility (“something I am supposed to do”; P1 and P3). Current patients of breast cancer (P2 and P5) mentioned that once they went through all the screenings, they got used to it and no longer gave it any thought.

Regarding barriers, participants mentioned: not experiencing any problems (P3), not being old enough for mammography (P4), or concerns that their breast size (both too big or too small) would not be ideal for screening (P1). Several participants (P2 and P10) pointed out the long waiting time for the screening appointment. For example, P2: *“*… So, I felt something about a year ago and then it took six months before I could get a screening.*”*

### The Screening Process

#### Interaction With Clinical Staff

Interaction with staff (technicians who perform the examination, doctors) emerged as a prominent topic of discussion and was perceived as a potential barrier to screening. Most of the participants emphasized the importance of good communication and sufficient explanation, as many of the participants liked the detailed explanation of the procedure in the video. Notably, some participants (P3 and P5) indicated that the quality of interaction and how well they were treated by medical staff is more important for them than the process of screening or test itself.

Participants across multiple focus groups (FG2, FG3, FG4, and FG5) attributed ideal qualities to clinical staff, including being friendly, kind, nice, empathetic, helpful, respectful, and positive. They emphasized the importance of being able to ask questions and get full, comprehensible information. The guidance and explanation before and during the procedure were also positively perceived.

Additional factors that could influence their interaction and level of comfort and trust during screening were the duration of their relationship with the doctor or clinical staff (P3, P5), the gender of the doctor or clinician (P4), and the doctor’s or clinical staff’s level of experience (P3). In addition, the inconsistency in seeing the same practitioner was noted as a potential factor affecting trust (P5).

##### Environment

Many participants (E3, P1, P2, P3, P4, P5, and P9) welcomed the idea of possible adjustments according to their needs, such as choice of music or podcast during procedure (P1, P4, and P5), choice of (informative) movie or short film playing on the screen (P2, P3, and P9), possibility to turn the lights on or off (E1) or having the option to wear an additional robe so as to feel less exposed (E1 and E6).

Participant E6 highlighted the importance of inclusivity, suggesting that focus and distraction methods should be adjusted to the individual’s needs. Furthermore, E6 recommended creating a less clinical atmosphere, advocating for a spa-like experience with sauna robes for increased comfort.

However, some participants (E6 and E5) expressed concerns that offering too many options might be overwhelming or trigger feelings of shyness during the procedure. Participants with previous screening experience, like P1 and P2, indicated they did not require extra care and prioritized efficiency over additional amenities.

Regarding exposure and privacy during the screening, some participants (P2, P4, and E6) were comfortable with nudity during the procedure, while others viewed it as a sensitive issue. Suggestions to maintain dignity included providing additional robes, curtains, or private rooms without interruptions from other staff.

##### Explanation

Most participants emphasized the importance of understanding the procedure’s steps and being informed about what occurs during the screening. They stressed the need to comprehend both the process beforehand and the ongoing events while undergoing the procedure. P7 specifically highlighted the significance of using clear and simple language during explanations. Participants 9 and 3 also stressed the value of enabling screeners to ask questions regarding the procedure and ensuring they receive satisfactory responses.

##### Time Issues

Some participants (P3 and P6) highlighted the importance of minimizing the duration of the procedure. They suggested that patients should be informed about the estimated length of the screening or any potential delays. For instance, participant P6 expressed frustration with the long time it took to receive the results. Participant P2 also pointed out that shorter procedures help alleviate discomfort.

### Postscreening Phase

#### Receiving Results

Preferences for receiving results varied, with some favoring a phone call for positive test results (by positive test result, we mean that cancer was found; P8 and P10), while others preferred email or text message for negative results (P8, P6, and P10). However, the consistency of the communication channel was deemed more crucial (P4 and P5), with a pre-agreed approach followed by doctors when conveying results to reduce stress.

Participants P2, P3, P10, and P11 highlighted the importance of face-to-face explanation and interpretation of results by experts. In addition, P1 and P2 suggested receiving a written summary of the results from the doctor during the meeting, facilitating retention of important information: “It would be nice to get some text you can remember afterwards... so you can tell your family” (P1).

### Perception of Thermography

#### Thermography Positives

In general, the first impression after viewing the video about AI-driven thermography was positive by all participants. They described the procedure as a good idea (P6 and P10), a game changer (P9), they liked the fact that the procedure is noninvasive, there is no breast manipulation, and it seems more comfortable (P2, P3, P4, P5, P8, P7, P9, P10, P11, E1, and E2) than the usual procedures they had already heard about. It was generally perceived as an “easier procedure” (P4 and P8), “better experience” (P11), and “less traumatic” (P4). Several participants (P1, P3, P6, P9, and E6) also mentioned the advantage of not using radiation.

#### Concerns About Thermography

Various concerns were raised in the focus groups regarding the new technology. One concern is its novelty, with participants expressing the need for clinical evidence to assess its effectiveness and reliability before fully trusting the procedure (P2, P3, P5, P8, P10, P11, and E6). Participants 2 and 3 also pointed out that the novelty of the machine can potentially affect other circumstances, such as the waiting time and the length of the procedure, for instance:

*Indeed this is one of the concerns that I would have… Is this a very expensive machine? Meaning that the hospital would have only one… Meaning, you'd have a longer waiting list than the current methodology. Um… so this definitely would be a concern like, because it seems to be taking longer, you know…*

[P2]

Some participants mentioned that being exposed in front of a camera could also be an issue for some women (P5, E1, E2, E3, E7, and E6). For example:

*I mean I think any screening device, especially like MRI, is really scary when you first look at it …you know to have these like cameras and then these fans, it might be a little bit overwhelming for some people as well.*

[P5]

Participants P2, P4, P5, E3, E5, E6, and E7 expressed a need for more information about data sharing, concretely what data are collected exactly and how is it processed and stored, for example:

*Even though it’s a heat sensor, it will be like a heat based picture of your [breasts]… So, I mean, I know it’s for health-related reasons, but it’s still a picture of your [breasts] even though it’s not like a normal picture.*

[E5]

Finally, many participants highlighted a cold temperature during the procedure as a possible problem (P1, P2, P5, P9, P10, P11, E4, and E7). As a possible solution, Participants 1 and 2 suggested showing a countdown of the cold air on the screen.

#### AI Perception

The use of AI was perceived rather neutrally (P4, P5, P9, and E5) to positively (P1, P2, P3, P6, P8, and P10); however, participants would like to have more information and explanations about how the AI in the thermography works (P2, P3, P4, and P11). Nevertheless, certain prerequisites for trusting thermography with AI were mentioned, such as training on a large data set (P2 and P8) and collaboration with a human expert; doctor (P2, P3, P5, P10, E1, and E4).

### Survey Insights Into Mammography: Motivators and Barriers

To complement the qualitative insights focused on thermography, this section summarizes the main survey findings about mammography motivators and barriers. A total of 268 responses were collected. After applying the exclusion criteria described in the methodology, 40 responses were removed. The final dataset used for analysis consisted of 228 eligible responses. The first part of the data (n=101) was analyzed in early September to produce preliminary results. The remaining responses (n=127) were added later, and the full dataset was analyzed together.

### Screening Experience and Demographics

Participants (N=228) were divided based on their experience, screeners (154/228, 68%, mean age 53 years, SD 12) and nonscreeners (74/228, 33%, mean age 36 years, SD 9.7). Most of the participants were European and currently lived in Europe, in the city and had a university degree. For more details about the socioeconomic characteristics, refer to Table 2 and Multimedia Appendix 2.

**Table 2.** Socioeconomic characteristics in the online survey.

Socioeconomic characteristics of the sample (N=228)	Responses, n (%)
Origins (European)	
Italy	127 (55.7)
Ireland	27 (11.8)
Lithuania	15 (6.6)
Belgium	13 (5.7)
Germany	5 (2.2)
Czech Republic	5 (2.2)
Spain	4 (1.8)
Netherlands	3 (1.3)
Portugal	3 (1.3)
Romania	2 (0.9)
Switzerland	2 (0.9)
Bulgaria	1 (0.4)
Croatia	1 (0.4)
Denmark	1 (0.4)
Greece	1 (0.4)
Norway	1 (0.4)
Montenegro	1 (0.4)
Slovakia	1 (0.4)
Sweden	1 (0.4)
Origins (outside Europe)	
United States of America	5 (2.2)
United Kingdom	4 (1.8)
Brazil	1 (0.4)
Congo	1 (0.4)
Pakistan	1 (0.4)
Peru	1 (0.4)
Philippines	1 (0.4)
Current country of residence	
European	224 (98.2)
Outside of Europe (United States of America, Australia)	4 (1.8)
Place of residence	
City	129 (56.6)
Rural areas	60 (26.3)
Towns and suburbs	39 (17.1)
Education	
University degree (bachelor’s or higher)	158 (69.3)
Upper-secondary education and post-secondary education	64 (28.1)
Lower secondary education	4 (1.8)
Primary education	2 (0.9)

### Awareness and Perceived Importance of Screening

Most women (146/228, 64%) were aware of available breast screening procedures near them. Nearly all participants (223/228, 98%) found breast screening important, which includes those who were not aware of their options. For more details, consult Multimedia Appendix 2.

### Sources of Information on Breast Cancer Screening

The most frequent sources of information about breast cancer screening among women who were aware of their options were doctors (122/146, 84%), family and friends (63/146, 43%), and internet (56/146, 38%). For full details, refer to Multimedia Appendix 2.

### Impact of Screening Invitations and Reminders

In total, 58% (132/228) of the participants had already received an invitation for breast cancer screening, and 88% of those who received the reminder (116/132) reported that it helped them to make an appointment for the screening, and most respondents (164/228, 72%) agreed that reminders would help them in the future to get a mammogram.

### Motivators and Barriers Towards Mammography

Personal values, such as belief in breast cancer prevention, were the main motivators (69/154, 45%), followed by an invitation from a screening program (68/154, 44%), a doctor’s recommendation (45/154, 29%) and family history of breast cancer (31/154, 20%; Table 3).

The most frequently chosen reason for nonscreeners was no recommendation from a doctor (39/74, 53%), no warning signals (27/74, 36%) or breast-related problems (21/74, 28%), and not a sufficient age for mammography (17/74, 23%). A total of 21 screeners out of 154 indicated the following barriers: pain during the procedure, the long wait for an appointment, radiation and invasiveness, fear of cancer, not sufficient evidence, high cost, and behavior of medical staff (Table 3). Most screeners did not indicate any physical issues that would prevent them from having mammography. Five respondents out of 154 mentioned specific obstacles, such as the size of their breasts and the inability to move their arm due to a frozen shoulder. Financial barriers were found to be insignificant factors in both groups, together with concerns related to language, cultural misunderstanding, and religious beliefs (consult Table A4 in the Multimedia Appendix 2 for more details). Regarding the procedure itself, mammography was perceived as less comfortable by people with actual experience (refer to Multimedia Appendix 2 for details).

**Table 3.** Survey results overview.

Motivators towards mammography (Screeners, n=154)	Responses, n (%)
What were the reasons for undergoing the mammography procedure? (multiple choice)	
I believe in breast cancer prevention	69 (44.8)
I got an invitation/reminder from a screening program	68 (44.2)
It was recommended by my doctor	45 (29.2)
I have/had breast cancer in the family	31 (20.1)
I had some warning signals	19 (12.3)
I have had a problem related to my breast before	13 (8.4)
I was advised to go from family members and/or friends	11 (7.1)
I had a positive experience in my last screening	5 (3.2)
My family members and/or friends go to the screening	5 (3.2)
Other (please specify)	8 (5.2)
Barriers towards mammography (Screeners, n=154)	
Was there anything that discouraged you from the mammography procedure?	
Yes	21 (13.6)
No	133 (86.4)
If yes, please specify what discouraged you? (Screeners, n=21; open question)	
Pain, discomfort	6 (29)
Long wait for the appointment, availability	5 (24)
Radiation	3 (14)
Fear	2 (10)
Not sufficient evidence	2 (10)
Too expensive	1 (5)
Behavior of medical staff	1 (5)
Other	1 (5)
Barriers towards mammography (Nonscreeners, n=74)	
What were the reasons why you did NOT undergo the mammography screening? (Nonscreeners, n=74)	
I did not get any recommendation from my doctor	39 (53)
I did not have any warning signals	27 (36)
I have not had any breast-related problem	21 (28)
I did not get any invitation/reminder	15 (20)
I do not have an overview if the screening is available for me	9 (12)
I was not advised to go from family members and/or friends	5 (7)
Mammography is painful	5 (7)
I do not have time for the mammography	3 (4)
I do not believe in breast cancer prevention	1 (1)
Mammography uses radiation	1 (1)
I do not know anyone with breast cancer	1 (1)
Other	22 (30)
Other reasons for not undergoing mammography (mentioned in the open questions, n=22)	
Age	17 (77)
Not important/did not think about it yet	2 (9)
Covid delay	1 (5)
Medical condition	1 (5)
No invitation	1 (5)

### Updated Breast Cancer Screening Journey

As described in the Methods section, survey and focus group insights were thematically mapped to the breast cancer screening journey using a collaborative Miro board. The updated journey reflects participant preferences and perceived barriers across the screening experience.

The revised journey is centered on a persona, a 45-year-old woman, reflecting the most commonly targeted age group for screening across European programs. Several key improvements were identified:

#### Before the screening

Participants emphasized the need for better preparation, including clear, accessible information on available screening options. Survey findings indicated that even women who valued screening often lacked awareness of what technologies were accessible to them. Flexibility in appointment scheduling was also considered important, with a preference for multiple booking channels such as online platforms, email, and phone.

#### Screening experience

Thematic analysis highlighted that empathetic interaction with clinical staff was a key factor influencing comfort and trust during the procedure. Participants valued being treated respectfully and receiving clear explanations throughout the process.

#### Postscreening phase

Receiving results in a preagreed format and timeframe was seen as essential. Focus group participants stressed the importance of structured communication, including both verbal and written explanations of results.

**Figure 4.**
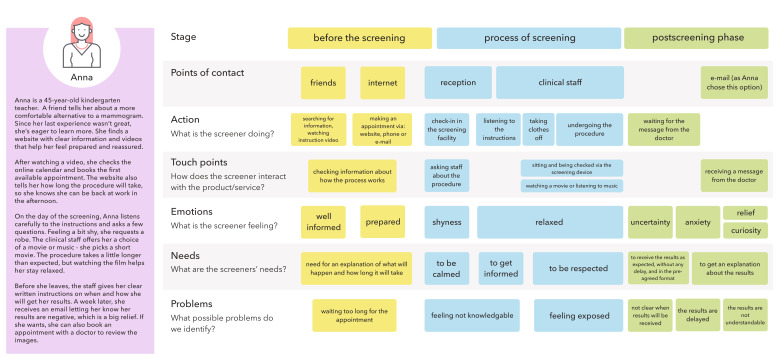
Proposed screening journey based on the insights from both qualitative and quantitative data.

These user-informed insights were integrated into a revised screening journey visual (Figure 4), intended to guide future patient-centered improvements in breast cancer screening programs.

## Discussion

### Principal Findings

This study explored women’s perception of ThermoBreast technology and their previous experiences with mammography and other breast cancer tests. Our findings suggest that personal values and doctor recommendations strongly influence participation, while discomfort, waiting times, and lack of awareness act as major barriers. Young age was also identified as a barrier, for which thermography could be a potential solution. In addition, while AI-driven thermography was perceived as a promising alternative, concerns about its novelty, accessibility, and reliability were raised. How the screener is treated during the procedure showed to be more important than the technology itself or physical discomfort that screening can bring. Collected insights were brought together to improve current breast cancer screening journeys and inspire future screening programs.

### Comparison With Previous Studies

Studies have consistently shown that discomfort is a major deterrent for mammography screening [17-19]. Although this was not prevalent in the survey results, women in the focus groups mentioned experiencing discomfort during the procedure as an important deterrent. Interestingly, our survey results indicate that women who had not yet undergone mammography perceived the procedure as more comfortable compared with those who had already experienced it. However, no significant difference was found.

Beyond physical discomfort, trust in health care providers plays a crucial role in screening participation. Similar to our findings, research by [10] has identified trust in health care providers as a crucial determinant of screening uptake, alongside effective communication [39]. In addition, our study highlights the importance of reminders and doctor recommendations as key motivational factors for screening participation. This aligns with previous research showing that structured reminders and physician encouragement significantly increase adherence to screening programs [40].

Cultural factors have been widely recognized as potential barriers to screening participation, particularly among minority populations. However, in contrast to previous studies that identified cultural, religious, and language barriers as significant obstacles [29,33], our findings did not indicate these as major factors influencing participation. This could be due to the specific demographic composition and the convenience nature of our sample, which may not fully represent minority groups who typically face these barriers. In addition, studies such as those by MacKinnon et al [27] and Foerster et al [28] have examined disparities in access to screening, particularly among underrepresented populations. Future studies should aim to include more diverse and underrepresented populations to ensure equitable and inclusive access to screening programs and emerging screening technologies.

While previous research has primarily examined the technical efficacy of thermography [17], our study uniquely contributes by capturing patient perceptions, concerns, and trust in AI-driven screening. Previous research, such as Ciria-Suarez et al [26], has mapped the breast cancer patient journey, highlighting logistical and emotional barriers to screening participation. Building on this, our study explores perceptions of thermography as an alternative screening method while also shifting the focus from the patient to the screener—an aspect that remains underexplored [27,28].

Finally, while previous research has investigated patient trust in AI-driven diagnostic tools [41], limited studies have specifically examined trust in AI-driven thermography. Our findings indicate that, while women appreciate the noninvasive nature of thermography, concerns persist regarding its reliability and clinical validation. This underscores the need for further research into the usability, accuracy, and integration of AI-driven thermography within existing screening programs.

### Limitations

Like other studies, this research has limitations. First, the sample was based on convenience rather than representativeness. The survey primarily served as a recruitment tool, with self-reported responses and no external validation. While we acknowledge that male breast cancer exists, this study focused on women, the majority of affected individuals. Younger women, not part of standard screening programs, were underrepresented despite efforts to include them. Language limitations may have excluded certain EU populations. Participants were mostly highly educated and engaged in breast cancer prevention, potentially narrowing perspectives on screening barriers. In addition, as focus groups were conducted in English, women with lower educational levels were underrepresented. Future research should seek broader inclusion.

Second, focus group insights were based on personal experiences and perceptions. While clinician interaction was a key factor, participants did not always specify health care roles they were referring to. Similarly, it was unclear whether discussed tests were part of initial screening or later diagnostics.

Third, as participants lacked real-life experience with ThermoBreast or other thermography techniques, direct comparison with mammography was not possible. However, insights into the procedure may inform other thermographic methods and contribute to a more user-centered screening approach. The proposed breast cancer screening journey offers valuable considerations for future programs, regardless of the technology used.

### Conclusions

AI-driven thermography holds promise as a noninvasive and radiation-free alternative to mammography, but its adoption depends on trust, clinical validation, and effective communication from health care professionals. Understanding the current mammography experience, including its barriers and motivators, provides valuable insights into how screening participation can be improved. Key areas for enhancement include regular screening invitations, efficient appointment scheduling, empathetic interactions with clinical staff, and clear, understandable explanations about the procedure, and structured communication of results.

While thermography could expand screening access for certain groups such as younger and immobile women, it does not eliminate all participation barriers. Some physical limitations, such as frozen shoulder, remain unaddressed. Furthermore, concerns about the reliability and accuracy of thermography highlight the need for further clinical validation. Our findings emphasize that the overall screening experience extends beyond the technology itself, with factors such as the clinical environment and the patient-provider relationship playing a crucial role in screening uptake. Nonetheless, many women welcomed the prospect of a less invasive and more accessible procedure.

Future research should prioritize making screening programs more inclusive, ensuring that underrepresented groups such as individuals with lower educational attainment, disadvantaged socioeconomic backgrounds, men, and younger women are better engaged. Tailoring the breast cancer screening journey to meet their specific needs will be essential for increasing awareness, accessibility, and participation in emerging screening technologies. Furthermore, more long-term studies on thermography, as well as direct comparisons between mammography and thermography, are needed to fully understand the differences in screening experiences and their impact on participation and outcomes.

## Supplementary material

10.2196/64954 Multimedia Appendix 1 Survey questions and focus group guide.

10.2196/64954 Multimedia Appendix 2 Recruitment materials and survey results.

## Abbreviations

AI: artificial intelligence
BC: breast cancer
CPE: Cancer Patients Europe
DBT: digital breast tomosynthesis
E: expert
MRI: magnetic resonance imaging
P: participant
