# Supplementary material for: Exploring Women’s Perceptions of Traditional Mammography and the Concept of AI-Driven Thermography to Improve the Breast Cancer Screening Journey: Mixed Methods Study
Source: JMIR Cancer. 2025 Sep 10;11:e64954. doi: 10.2196/64954 (PMC12422528; doi:10.2196/64954)
Supplement: Multimedia Appendix 1 [file cancer-v11-e64954-s001.docx]

**List of survey questions:**

- Q0. What is your sex?
- Woman
- Man
- Other

- Q1. Do you find regular breast screening important?
- Definitely yes
- Rather yes
- Rather not
- Definitely not
- I do not know

- Q2. With what breast cancer screening procedures below are you familiar? [Awareness]
- Mammography = A method using a special X-ray machine that produces.
- Ultrasound = A method that provides an image using sound signals to identify breast glands and possible tumours.
- Breast Magnetic Resonance Imaging (MRI) = An examination that uses radio waves and strong magnets to make pictures of the inside of the breast.
- Tomosynthesis (3D Mammography) = A different version of digital x-ray mammogram which creates 2D and 3D-like pictures of the breasts.
- Clinical examination = Using the pads of the fingers, your healthcare provider checks your entire breast, underarm, and collarbone area for any lumps or abnormalities.
- None of the above

- Q3. Do you know what breast screening procedures are available near you? [Availability]
- Yes
- No
- I am not sure

- Q4. I learnt about breast cancer screening from:
- Newspapers
- TV programmes
- Books/magazines
- Internet
- Brochures
- Family/friends
- Public health forums
- Doctors (general practitioner, gynaecologist,…)
- Other
- Q5. Have you ever received an invitation or a reminder for breast cancer screening?
- Yes
- No
- I do not remember

- Q6. Did the invitation or a reminder has prompted you to make an appointment for breast cancer screening?
- Definitely yes
- Rather yes
- Rather not
- Definitely not
- I do not remember
- Q7. Have you already had an experience with a mammography procedure?
- Yes
- No

- Q8. When did you have your last mammography procedure?
- Within the past 2 years
- More than 2 years ago
- I do not remember

- Q9. What were the reasons for undergoing the mammography procedure?
- It was recommended by my doctor
- I got an invitation/reminder from a screening programme
- I was advised to go from family members and/or friends
- I had some warning signals
- I have/had breast cancer in the family
- My family members and/or friends go to the screening
- I have had a problem related to my breast before
- I have had a positive experience in my last screening
- I believe in breast cancer prevention
- Other (please specify)

- Q10. Were there any physical obstacles that made the mammography procedure difficult for you? (for example: not able to stand during the screening)
- Yes, please specify
- No
- I do not remember

- Q11. Please indicate to what extent you agree with the following statements (Strongly disagree – Disagree – Neither agree or disagree – Agree – Strongly agree):
- Having a routine mammography makes me worry
- Having a mammography is embarrassing
- Having a mammography takes too much time
- Having a mammography is painful

- Q12. Please indicate to what extent you agree with the following statements (Strongly disagree – Disagree – Neither agree or disagree – Agree – Strongly agree):
- Having a mammography is not comfortable
- Having a mammography is too expensive
- I was afraid that the doctor would not understand my language
- I was afraid that the doctor would not understand my culture
- Having mammography is not align with my religious beliefs
- I believe in breast cancer prevention

- Q13. Was there anything that discouraged you from the mammography procedure?
- Yes - please specify
- No

- Q14. What were the reasons why you did NOT undergo the mammography screening?
- I did not get any recommendation from my doctor
- I did not get any invitation/reminder
- I was not adviced to go from family members and/or friends
- I do not know anyone with breast cancer
- I did not have any warning signals
- I have not had any problem related to my breast
- I do not have time for the mammography
- I do not have an overview if the screening is available for me
- I do not believe in breast cancer prevention
- Mammography uses radiation
- Mammography is painful
- Other

- Q15. Are there any physical obstacles that would make the mammography procedure difficult for you? (for example: not able to stand during the screening)
- Yes - please specify
- No
- I do not remember

- Q16. Please indicate to what extent you agree with the following statements (Strongly disagree – Disagree – Neither agree or disagree – Agree – Strongly agree):
- Having a routine mammography would make me worry
- Having a mammography would be embarrassing
- Having a mammography would take too much time
- Having a mammography would be painful

- Q17. Please indicate to what extent you agree with the following statements (Strongly disagree – Disagree – Neither agree or disagree – Agree – Strongly agree):
- Having a mammography would not be comfortable
- Having a mammography would be too expensive
- I am afraid that doctor would not understand my language
- I am afraid that the doctor would not understand my culture
- Having mammography would not be align with my religious beliefs
- I believe in breast cancer prevention

- Q18. Please indicate to what extent you agree with the following statements (Strongly disagree – Disagree – Neither agree or disagree – Agree – Strongly agree):
- Reminder letters  would  help  me  to  get  a  mammogram
- Reminder phone calls  or  text  messages  would  help  me  to  get  a  mammogram
- Routine educational talks regarding breast cancer awareness would help me to get a mammogram
- I feel confident that if I had a mammogram done, any abnormalities in my breasts will be detected
- I can arrange other things in my life to get a mammogram

- Q19. Now, we would like to ask you a couple of personal questions. What is your year of birth (e.g. 1973)?
- [Open question]

- Q20. What is your highest obtained degree?

- No formal education or below primary education
- Primary education
- Lower secondary education
- Upper secondary education
- Post-secondary education
- Bachelor's or equivalent
- Master's or equivalent
- Doctoral or equivalent
- Q21. Which country are you from?
- [Select from list of all countries]

- Q22. In which country do you currently live?
- [Select from list of all countries]
- Q23. How would you describe the place where you live?
- City
- Town or suburb
- Rural area (village)

**Focus group guide**

I. Introduction [15 min]

- Introduction and explaining the purpose of the project + recording [5 min]
- Introduction of participants [10 min]
- Brief introduction per participant, start this by as a facilitator introducing yourself in that way
- Age, country of residence, experience with screening (type of screening and reason to go/reason not to go)
- What I liked most about the screening I went through and what i hated most
- What is their experience, and why did they undergo the test, do you perceived yourself in a risk

1. Presenting thermography [15 min]

- Showing the video about thermography and explain that the technology is under a clinical trial [5 min]
- First impression and comparison to other procedures [10 min]
- What is your first impression of the ThermoBreast procedure?
- What do you think having thermography would be like? What thoughts would be going through your head if you were going to have one? What sort of questions would you ask about it?
- Imagine that this introduction movie would be shown for you as a preparation for a procedure next week. What would you think?
- What feelings do you think you would have?
- Are there any worries that come to mind?
- How do you feel about the fact that the ThermoBreast is using Artificial Intelligence?

1. Comfort during the procedure [10 min]

- Would you consider undergoing the ThermoBreast procedure? Why? What, on the contrary, discourages you from the procedure? [Would it help if there was a nurse with you? Special cloth to minimize your exposure? Curtain?]
- Is there anything still missing that would make you feel more comfortable during the thermography procedure?
- If you think about the other breast cancer screening procedures that you know about, what procedure do you perceive as more comfortable?

1. Trust in the technology [10 min]

- Do you have a feeling of trust in the ThermoBreast procedure? Does it seem safe to you? If not, can you describe why?
- What sort of reassurances would you need about the process to trust the technology?
- Is there anything unclear/hard to understand about the ThermoBreast procedure? If so, what information is missing? How would you like to receive it and from whom? [A video, a text or an explanation by a professional?]

1. After the screening [10 min]

- After you participate in the screening, how would you like to be approached about the results? (message, phone call, meeting with a doctor,...)
- What information would you like to have? (only the results, or also the thermography images with an explanation?) Who would you like to consult?

1. Closing [5 min]

Anything you want to add that was not discussed?

Thanking participants and informing them about next steps (results from the study etc.)
