# Supplementary material for: Exploring Women’s Perceptions of Traditional Mammography and the Concept of AI-Driven Thermography to Improve the Breast Cancer Screening Journey: Mixed Methods Study
Source: JMIR Cancer. 2025 Sep 10;11:e64954. doi: 10.2196/64954 (PMC12422528; doi:10.2196/64954)
Supplement: Multimedia Appendix 2 [file cancer-v11-e64954-s002.docx]

**Appendix II**

**Social media posts for recruitment**


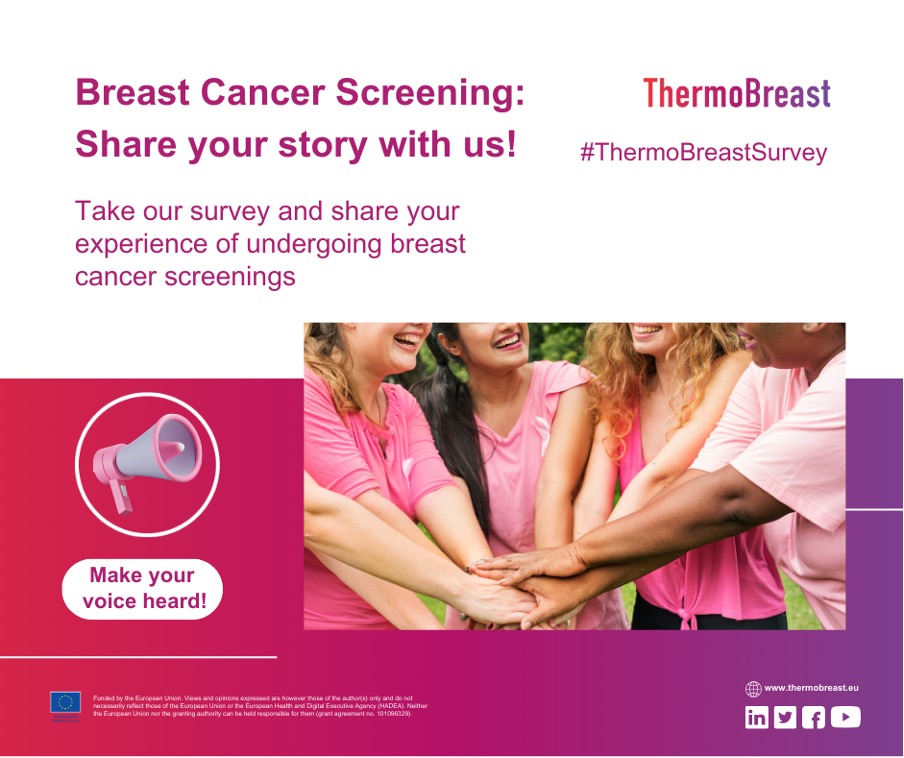


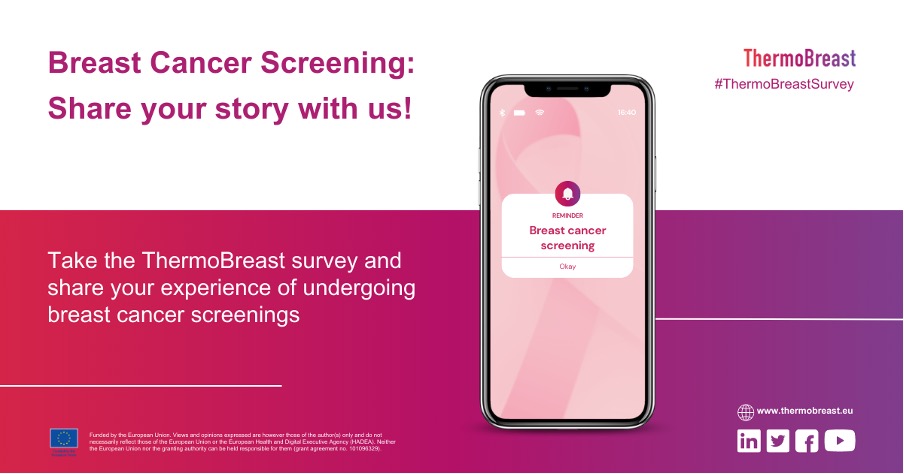


| **Table A0: Socio-economical Characteristics of the Sample (N=228)** | | | | |
| --- | --- | --- | --- | --- |
| **Average age** |  | 47,2 (SD=12) |  |  |
| Screeners |  | 53 (SD=8.8) |  |  |
| Non-screeners |  | 36 (SD=9.7) |  |  |
| **Origins** |  |  | Percentages (%) |  |
| European |  | 94% (214 participants out of 228) |  |  |
| Outside Europe (Congo, Pakistan, Peru, Philippines, UK, USA) |  | 6% (14 out of 228) |  |  |
| **Current country of residence** |  |  |  |  |
| European |  | 98% (224 out of 228) |  |  |
|  |  |  | Italy | 55% (126 out of 228) |
|  |  |  | Belgium | 11% (25 out of 228) |
|  |  |  | Ireland | 11% (26 out of 228) |
|  |  |  | Lithuania | 7% (15 out of 228) |
|  |  |  | Czech Republic | 3% (6 out of 228) |
|  |  |  | Germany | 3% (6 out of 228) |
|  |  |  | Switzerland | 2% (5 out of 228) |
|  |  |  | France | 1% (3 out of 228) |
|  |  |  | Portugal | 1% (3 out of 228) |
|  |  |  | Spain | 1% (3 out of 228) |
|  |  |  | Netherlands | 1% (2 out of 228) |
|  |  |  | Austria | 1% (1 out of 228) |
|  |  |  | Bulgaria | 1% (1 out of 228) |
|  |  |  | Croatia | 1% (1 out of 228) |
|  |  |  | Denmark | 1% (1 out of 228) |
| Outside Europe (USA, Australia) |  | 2% (4 out of 228) |  |  |
|  |  |  | US | 1% (3 out of 228) |
|  |  |  | Australia | 1% (1 out of 228) |
| **Place of residence** |  |  |  |  |
| City |  | 57% (129 out of 228) |  |  |
| Rural areas |  | 26% (60 out of 228) |  |  |
| Towns and suburbs |  | 17% (39 out of 228) |  |  |
| **Education** |  |  |  |  |
| University degree (bachelor’s or higher) |  | 69% (158 out of 228) |  |  |
| Upper-secondary education and post-secondary education |  | 28% (64 out of 228) |  |  |
| Lower secondary education |  | 2% (4 out of 228) |  |  |
| Primary education |  | 1% (2 out of 228) |  |  |

| **Table A1: Barriers towards mammography – comparisons of screeners vs non-screeners** | | | | |
| --- | --- | --- | --- | --- |
| **Having a mammography is too expensive** (n=227) | N | Agree (%) | Neither agree or disagree (%) | Disagree (%) |
| Screeners | 153 | 23 | 34 | 96 |
| Non-screeners | 74 | 5 | 25 | 44 |
| All women | 227 | 28 | 59 | 140 |
|  | N | Mean (SD) | t(df) | p-value |
| Screeners | 153 | 2.18 (1.16) | -0.139 (177.08) | 0.890 |
| Non-screeners | 74 | 2.20 (0.92) |  |  |
| **I was afraid that the doctor would not understand my culture** | N | Agree (%) | Neither agree or disagree (%) | Disagree (%) |
| Screeners | 153 | 3 | 20 | 130 |
| Non-screeners | 74 | 1 | 2 | 71 |
| All women | 227 | 4 | 22 | 201 |
|  | N | Mean (SD) | t(df) | p-value |
| Screeners | 153 | 1.54 (0.79) | 2.25 (170.83) | 0.026 |
| Non-screeners | 74 | 1.31 (0.66) |  |  |
| **I was afraid that the doctor would not understand my language** |  | Agree (%) | Neither agree or disagree (%) | Disagree (%) |
| Screeners | 153 | 3 | 21 | 129 |
| Non-screeners | 74 | 0 | 2 | 72 |
| All women | 227 | 3 | 23 | 201 |
|  | N | Mean (SD) | t(df) | p-value |
| Screeners | 153 | 1.56 (0.80) |  |  |
| Non-screeners | 74 | 1.28 (0.51) |  |  |
| **Having mammography is not align with my religious beliefs** | N | Agree | Neither agree or disagree | Disagree |
| Screeners | 153 | 1 | 12 | 140 |
| Non-screeners | 74 | 0 | 2 | 72 |
| All women | 227 | 1 | 14 | 212 |
|  | N | Mean (SD) | t(df) | p-value |
| Screeners | 153 | 1.29 (0.64) | 0.857 (225) | 0.393 |
| Non-screeners | 74 | 1.22 (0.48) |  |  |

| **Table A2: Awareness and Attitude Towards Screening** | | | | |
| --- | --- | --- | --- | --- |
| **Do you know what breast screening procedures are available near you?** | N | Yes | No | I am not sure |
| Screeners | 154 | 112 | 13 | 29 |
| Non-screeners | 74 | 34 | 19 | 21 |
| Total | 228 | 146 | 32 | 50 |
|  |  |  |  |  |
| **I learnt about breast cancer screening from:** ^[[1]](#footnote-1)^ (multiple choice) | Percentages (%) |  |  |  |
| Doctors (general practitioner, gynaecologist,...) | 84% (122 out of 146) |  |  |  |
| Family or friends | 43% (63 out of 146) |  |  |  |
| Internet | 38% (56 out of 146) |  |  |  |
| Public health forums | 21% (30 out of 146) |  |  |  |
| TV programmes | 17% (25 out of 146) |  |  |  |
| Newspapers | 15% (22 out of 146) |  |  |  |
| Brochures | 14% (21 out of 146) |  |  |  |
| Books/magazines | 13% (19 out of 146) |  |  |  |
| Other | 8% (11 out of 146) |  |  |  |
| **Do you find regular breast screening important?** | N | Yes | No | I am not sure |
| Screeners | 154 | 152 | 1 | 1 |
| Non-screeners | 74 | 71 | 3 | 0 |
| Total | 228 | 223 | 4 | 1 |

| **Table A3: Screening experience** | | | | |
| --- | --- | --- | --- | --- |
| **Having a mammography is not comfortable (own question)** | N | Agree | Neither agree or disagree | Disagree |
| Screeners | 153 | 63 | 22 | 68 |
| Non-screeners | 74 | 15 | 12 | 47 |
| Total | 227 | 78 | 34 | 115 |
|  | N | Mean (SD) |  |  |
| Screeners | 153 | 2.91 (1.35) |  |  |
| Non-screeners | 74 | 2.31 (1.22) |  |  |
| **Mammography perception – scale**  (example of items: Having mammography is embarassing, Having a mammography is painful) | N | Agree | Neither agree or disagree | Disagree |
| Screeners (n=154) | 154 | 13 | 41 | 100 |
| Non-screeners (n=74) | 74 | 4 | 17 | 53 |
| Total | 228 | 17 | 58 | 153 |
|  | N | Mean (SD) |  |  |
| Screeners | 154 | 2.25 (0.87) |  |  |
| Non-screeners | 74 | 2.12 (0.86) |  |  |
|  |  |  |  |  |

| **Table A4: Prevention** | | | | |
| --- | --- | --- | --- | --- |
|  | | | | |
| **Have you ever received an invitation or a reminder for breast cancer screening?** | N | Yes | No | I do not remember |
| All women | 228 | 132 | 91 | 5 |
| **Did the invitation or a reminder has prompted you to make an appointment for breast cancer screening? (women who responded yes)** | N | Yes (definitely yes + rather yes) | No  (Rather not + definitely not) |  |
| Women who responded yes to receiving a reminder | 132 | 116 | 16 |  |
| **Cues to action scale [Mohamed] (example of items:** Reminder letter would help me to get a mammogram”, “Routine educational talks regarding breast cancer awareness would help me to get a mammogram”) |  | Agree | Neither agree or disagree | Disagree |
| Screeners | 154 | 109 | 34 | 11 |
| Non-screeners | 74 | 55 | 14 | 5 |
| Total | 228 | 164 | 48 | 16 |
|  | N | Mean (SD) |  |  |
| Screeners | 153 | 2.64 (0.61) |  |  |
| Non-screeners | 74 | 2.68 (0.60) |  |  |

1. Only for respondents who answered “yes” to the question: “Do you know what breast screening procedures are available near you?” [↑](#footnote-ref-1)
